# Polymorphism of glutathione S-transferase in the population of Polish patients with carcinoma of the prostate

**DOI:** 10.1007/s11356-020-08435-7

**Published:** 2020-03-24

**Authors:** Joanna M. Drozdz-Afelt, Beata Koim-Puchowska, Grzegorz Klosowski, Piotr Kaminski

**Affiliations:** 1 Department of Biotechnology, Kazimierz Wielki University, Księcia Józefa Poniatowskiego St.12, PL, 85-671 Bydgoszcz, Poland; 2 Department of Ecology and Environmental Protection, Collegium Medicum in Bydgoszczy, Nicolaus Copernicus University in Toruń, M. Curie Skłodowskiej St.9, PL, 85-094 Bydgoszcz, Poland

**Keywords:** Glutathione S-transferase, Glutathione S-transferase M1, Glutathione S-transferase P1-1, Glutathione S-transferase T1, Polymorphism, Prostate cancer

## Abstract

The aim of the study was frequency analysis of *GSTM1*, *GSTT1*, and *GSTP1* polymorphisms of glutathione S-transferase in the group of patients with prostate cancer and in a control group of healthy individuals. Genomic DNA was isolated; molecular analysis of glutathione S-transferase *M1* and *T2* polymorphisms was performed using multiplex PCR and RFLP methods. The products of the PCR reaction were then visualized in agarose gel, and a statistical analysis of the results was performed. No statistically significant differences were found in the frequency of glutathione S-transferase polymorphisms between 66 patients with prostate cancer and the control group (64 healthy volunteers). The *GSTM1* gene deletion was found in ca. 47% of patients with prostate cancer and in ca. 55% of the controls. The *GSTT1* deletion was found in approximately 17% of patients and 14% of the controls. The distribution of *GSTP1 Ile/Ile*, *Ile/Val*, and *Val/Val* polymorphisms was ca. 51.5%, 39%, and 9% in the group of patients and 61%, 34%, and 5% in the control group, respectively. The results indicate that there is no relationship between glutathione S-transferase polymorphisms and prostate cancer in the study group, which is a novelty when compared with the previous work on the role of these genetic variants in the etiology of cancer.

## Introduction

Over the last three decades, the incidence of malignant tumors has increased dramatically. Oncologists cite data showing that the diagnosis of cancer has doubled in Polish patients. Cancers are also the second leading cause of death. They are characterized by increasing incidence rate, ca. 1.96% on an annual basis in men in Poland (Didkowska et al. 2009; Wojtys et al. 2013; Didkowska and Wojciechowska 2015; Osowiecka et al. 2019).

Prostate cancer is currently one of the most common cancers, and the mortality associated with it is one of the highest among men, second only to lung cancer. Until the 1980, the incidence rate was dramatically increasing. After the introduction of screening for prostate-specific antigen (PSA), the incidence rate of prostate cancer stopped rising, remaining at a stable level (Kral et al. 2011; Osowiecka et al. 2019).

Despite the progress in understanding the basis of the disease, which includes the discovery of the role of testosterone, ongoing research is aimed at determining both environmental and genetic factors that increase the susceptibility to this disease (Ricks-Santi et al. 2010; Osowiecka et al. 2019).

Prostate cancer is the second most common malignant tumor in men which is most commonly seen in older adults. Over 80% of prostate cancer cases are diagnosed in men aged 65 or older (Daniyal et al. 2014), and the risk of developing the disease increases after the age of 50 (Nurzyński 2011). Prostate cancer mortality is low (10%), but it is believed that the disease often remains undetected. Because of its high prevalence, health consequences, and mortality, prostate cancer has become one of the major health problems of the modern world (Daniyal et al. 2014).

Until now the etiology of the disease has not been clearly defined despite the recognition of well-established risk factors, such as age (the main risk factor), ethnic and geographical origin, and family history of prostate cancer (Sivonová et al. 2009; Daniyal et al. 2014; Mandair et al. 2014; Szot et al. 2014). It is believed that environmental factors are responsible for the development of prostate tumors, but an important role is also attributed to genetic factors (Kral et al. 2011).

Studies showed that a patient whose father or brother suffered from prostate cancer has a 2-fold increased risk of developing the disease compared with the general population (Sivonová et al. 2009). Men with one, two, or three first-degree relatives diagnosed with prostate cancer have, respectively, two-fold, five-fold, and eleven-fold increased risk of developing the disease, compared with those who did not report any history of familial prostate cancer (Zeigler-Johnson et al. 2008). Scandinavian studies involving twins suggested that inheritance of predisposing genes is responsible for 42% of prostate cancer cases; the remaining part is dependent on environmental factors (Wang et al. 2018).

The etiology of prostate cancer includes factors that the man was exposed to at home or at work (Nurzyński 2011). Recent studies highlight the impact of factors such as smoking, excessive alcohol consumption, high-fat diet, sexual behavior, and exposure to UV radiation or to carcinogenic substances (Sivonová et al. 2009; Daniyal et al. 2014; Szot et al. 2014). Occupational exposures include contamination with substances of industrial origin but also with pesticides used in agriculture (Szot et al. 2014).

Recent work focuses on gene polymorphisms that may be responsible for the increased susceptibility to prostate cancer (Ricks-Santi et al. 2010; Mittal et al. 2011). A number of polymorphisms responsible for the increase in the incidence of prostate cancer have been identified, and selected mutations in the *CHECK2*, *NBS1*, and *BRCA1* genes are analyzed as markers. In addition, new population-specific changes that may increase the risk of prostate cancer are being sought (Mittal et al. 2011; Giri and Beebe-Dimmer 2016; Pritchard et al. 2016; Wang et al. 2018).

Studies on the identification of genetic polymorphisms of glutathione S-transferase (GST), one of the major enzymes involved in the inactivation of carcinogens, are being vigorously pursued. Due to their wide substrate specificity, glutathione S-transferases are an interesting research topic. Currently, extensively studied are GST polymorphisms that cause reduction or loss of enzymatic activity, i.e., *GSTM1*, *GSTT1*, and *GSTP1*. Reports on relationships between these genetic variants and neoplastic changes suggest their involvement in the etiology of colon, breast, lung, and prostate cancer. Despite the ambiguous results, it is suggested that the changes in the human glutathione S-transferase gene may be linked to the incidence of various types of cancer (Lavender et al. 2009; Chatterjee and Gupta 2018).

Glutathione S-transferases belong to a multigenic family that is responsible for the production of enzymes of phase II of xenobiotic metabolism (Mo et al. 2009; Kwon et al. 2011; Giri and Beebe-Dimmer 2016). These proteins catalyze the coupling of glutathione with electrophilic components mediating the removal of endogenous and exogenous substances from the body, including carcinogens (Lavender et al. 2009).

For example, GST enzymes are involved in the inactivation of such carcinogens as polycyclic aromatic hydrocarbons (PAHs), which are components of cigarette smoke, grilled meats, and diesel exhaust. GSTs are also involved in the detoxification of reactive oxygen species and endogenous steroid hormone metabolites (Nock et al. 2009). Other GST functions include modulation of various enzymes, e.g., those important for DNA repair (Mo et al. 2009).

GST enzymes are encoded by at least 8 distinct loci, each containing one or more homodimeric or heterodimeric isoforms. *GSTT1*, *GSTM1*, and *GSTP1* belong to the best-studied loci (Mo et al. 2009). *GSTT1* and *GSTM1* are deletion variants resulting in the loss of enzymatic activity. *GSTP1* is a polymorphism causing substitution inside the active site of the enzyme that leads to changes in conjugation activity and substrate-specific thermostability (Lavender et al. 2009; Benabdelkrim et al. 2018).

GST enzymes are primarily involved in the metabolism of xenobiotics including various potential carcinogens. Therefore, it is believed that they play a role in protecting the organism from toxic and carcinogenic components that get into the body in the form of contaminants, drugs, or food additives. It is suggested that people with loss-of-function genes or genes for altered enzymatic activity may have limited capacity to efficiently eliminate carcinogens, which may lead to their accumulation and consequently to an increased risk of mutations (Kwon et al. 2011; Benabdelkrim et al. 2018).

*GSTM1* gene encodes a glutathione S-transferase that belongs to the *mu class*. The function of this enzyme is the detoxification of electrophilic substances such as drugs, various types of carcinogens (benzopyrene—a component of cigarette smoke), or products of oxidative stress. This is done by coupling with glutathione, in phase II of metabolism of xenobiotics (http://www.ncbi.nlm.nih.gov/gene/2944, Kwon et al. 2011; Weich et al. 2017).

It has been shown that the inheritance of the *GSTM1* deletion, i.e., the homozygous *GSTM1* null genotype, leads to inactivity of this form of the enzyme. It is believed that the null genotype has a reduced ability to detoxify some carcinogens and therefore may be associated with an increased risk of solid tumors (Sivonová et al. 2009; Kwon et al. 2011; Weich et al. 2017).

According to Mo and co-workers 2009, the frequency of this polymorphism in Caucasians ranges from 13.1 to 54.5%. Slovak researchers reported that the frequency of *GSTM1* polymorphism among white Europeans ranges from 47 to 58% (Sivonová et al. 2009).

The *GSTT1*-theta 1 gene located on chromosome 22 (locus 22q11.23) is also a member of the superfamily of genes encoding proteins that catalyze the coupling of reduced glutathione with various hydrophobic and electrophilic compounds. This gene is probably involved in carcinogenesis processes. The function of *GSTT1* is the detoxification of smaller, reactive hydrocarbons, e.g., ethylene oxide (http://www.ncbi.nlm.nih.gov/gene/2952, Kwon et al. 2011).

The *GSTT1* null genotype leads to the lack of enzymatic activity of the protein, as in the case of the *GSTM1* null polymorphism. Due to the reduced detoxification capacity of ethylene oxide metabolites, this variant can provide information on exposure to environmental or nutritional factors that can damage genetic material (Safarinejad et al. 2011; Kwon et al. 2011).

*GSTT1* deletion is associated with an increased risk of developing ovarian, bladder, or lung cancer. The frequency of homozygous deletion of this gene was estimated at 11.1–28.6% in Caucasians (Mo et al. 2009), while the Slovak study reports 13–25% in white Europeans (Sivonová et al. 2009).

GSTP1 is one of the major enzymes involved in the inactivation of carcinogenic substances found in cigarettes and cigarette smoke. Substances neutralized by GSTP1 include epoxide benzo-(alpha)-pyrene diol or acrolein, a compound that is a frequent environmental pollutant and exhibits a high reactivity with the cells (Harries et al. 1997; Safarinejad et al. 2011). It was also found that the gene coding for glutathione S-transferase is inactivated by hypermethylation at the early stages of prostate carcinogenesis and that its expression is also disturbed in samples of other cancers, which may suggest its role in the etiology of neoplastic diseases (Harries et al. 1997).

The best studied polymorphism *GSTP1 Ile105Val,* resulting from substitution of 105 Ile codon for Val codon, most probably leads to changes in the substrate-specific thermostability and affects the conjugation activity. When compared with the *GSTP1 Ile/Ile* variant, the *GSTP1 Val/Val* variant was associated with reduced detoxification of epoxy diols of certain polycyclic aromatic hydrocarbons (Buchard et al. 2007; Lavender et al. 2009; Shin et al. 2016).

According to meta-analysis of Mo et al. (2009), the frequency of this polymorphism, located at 11q13, for the *Val/Val* homozygous variant is about 9–12.6% in Caucasians. Sironova and co-workers 2009 cited the data on the frequency of heterozygotes *Ile/Val* and homozygotes *Val/Val* in the population of white Europeans, which was between 38 and 45.7% and between 7 and 13%, respectively (http://www.ncbi.nlm.nih.gov/gene/2950).

The aim of this study was to analyze the frequency of polymorphic glutathione S-transferase (*GSTM1, GSTT1, GSTP1*) in patients with prostate cancer compared with the control group of healthy men from Poland. The polymorphisms listed above are an interesting research topic in the context of cancer. Prostate cancer is a multifactorial disease caused by genetic and environmental factors. Identification of carcinogens and genes responsible for prostate cancer susceptibility is an interesting topic for further studies aimed at a better understanding of the etiology of the disease.

## Materials and methods

In the study, we used blood samples collected from 66 patients of the Oncology and Brachytherapy Department of the Oncology Center in Bydgoszcz with diagnosed prostate cancer and from 64 healthy volunteers recruited at the outpatient clinic (SP ZOZ) in Mogilno and the Department of Prevention and Health Promotion of the Oncology Center in Bydgoszcz.

Men with a recent blood transfusion were excluded from both the cancer group and the control. The control group consisted of men over 50 years old, so that the age of the sick and the controls was similar. Men were qualified to the control group based on surveys. First of all, their health was taken into account. In particular, the candidates had to meet the following requirements:

1. No diagnosed cancer history

2. No major surgical procedures

3. The results of PSA tests in the reference range (up to 4 ng*ml^−1^) and normal prostate on digital rectal examination

The work was approved by Ethical Committee of Collegium Medicum in Bydgoszcz (KB 65/2012; consent dated February 28, 2012, and the relevant annexes).

The material for analysis was blood collected from the ulnar vein. For blood collection, we used 2-ml hematology tubes containing K_3_EDTA.

In order to obtain separated serum, blood samples were centrifuged (2000 ×*g*, for 15 min, at 4 °C), and the material needed for analysis was transferred to Eppendorf tubes. All tubes with test material were stored at − 80 °C until the planned analyzes were performed. DNA isolation from the blood was carried out using Master Pure™ DNA Purification Kit for Blood (Epicenter Biotechnologies, USA).

### *GSTM1* and *GSTT1* polymorphism analysis using multiplex PCR

The material isolated from blood was amplified using the multiplex PCR method according to Gara et al. (2010). This method provides information on both deletions (*GSTM1* and *GSTT1*) in one reaction.

The reaction solution contained 50 ng DNA, 1 μl 200 nmol starters, 4 μl 25 mM MgCl_2,_ 5 μl reaction buffer (10×), 1 μl 10 mM dNTPs, and 2 U Taq polymerase. The volume of the solution was adjusted with water to 50 μl.

Three pairs of starters were used:

- *GSTM1*- sense G1 (GAACTCCCTGAAAAGCTAAAGC)
- *GSTM1*- anti G2 (GTTGGGCTCAAATATACGGTGG)
- *GSTT1*- sense T1 (TTCCTTACTGGTCCTCACATCTC)
- *GSTT1*- anti T2 (TCACCGGATCATGGCCAGCA)
- *Albumin*- sense A1 (GCCCTCTGCTAACAAGTCCTAC)
- *Albumin*- anti A2 (GCCCTAAAAAGAAAATCGCCAATC)

Multiplex PCR proceeded according to the following protocol: DNA initial denaturation at 94 °C for 5 min, initial hybridization at 64 °C for 2 min, 30 cycles of elongation for 30 s at 72 °C, denaturation for 20 s at 94 °C, hybridization for 20 s at 64 °C, and then final extension for 7 min at 72 °C.

The resulting DNA fragments were then separated in a 1.5% agarose gel. The gels were stained with Midori Green reagent (a noncarcinogenic alternative to ethidium bromide) and run at 100 V for about 50 min. The results were then visualized in a chamber equipped with a camera and UV lamp. pBR322 DNA/Alu I was run on the gel as a size marker.

The lack of amplification of 215 bp and 480 bp fragments indicated the presence of deletions in the *GSTM1* and *GSTT1* fragments, respectively. A fragment of albumin gene (380 bp) was used as a positive reaction control.

### *GSTP1* polymorphism analysis using PCR-RFLP

Genetic material isolated from blood was subjected to PCR-RFLP, which allows to detect a given change in DNA by using an appropriate restriction enzyme. PCR and digestions were carried out according to the method developed by Harries et al. (1997).

The reaction solution contained 50 ng DNA, 1 μl 200 nmol starters, 2.4 μl 25 mM MgCl_2,_ 4 μl reaction buffer (10×), 1 μl 10 mM dNTPs, and 2 U Taq polymerase. The volume of the solution was adjusted with water to 40 μl.

The following pair of starters was applied:

- P105F (ACC CCA GGG CTC TAT GGG AA)
- P105R (TGA GGG CAC AAG AAG CCC CT)

The obtained fragments were then digested with the restriction enzyme Alw26I using 20 μl of PCR product, 4 U of ALw26I, 2.5 μl of Tango buffer (10×), and 2 μl of H_2_O. The reaction was carried out for 2 h at 37 °C. The resulting DNA fragments were then separated in a 4% agarose gel at 110 V for ca. 30 min. The gels were stained with Midori Green reagent. The results were visualized in a chamber equipped with a camera and UV lamp. pUC/Msp I was run on the gel as a size marker. A 176-bp band on the agarose gel indicated the presence of the *Ile/Ile* polymorphism, two bands of 91 bp and 85 bp corresponded to the *Val/Val* polymorphism, while the presence of all three bands in the gel path (176 bp, 91 bp, and 85 bp) indicated the presence of a heterozygous variant of the *GSTP1 Ile/Val* gene.

Statistical analysis was performed with STATISTICA 10 software for Windows 10 using descriptive statistics and statistical significance tests. In the first step, Shapiro-Wilk normality test was used to determine if the analyzed data were normally distributed. As the null hypothesis about the normality of the distribution was rejected for variables used in this work, between-group differences were analyzed using the Mann-Whitney U test. For all *analyzes a significance level* of 5% (*p* < 0.05) was adopted.

## Results

**Fig. 1:**
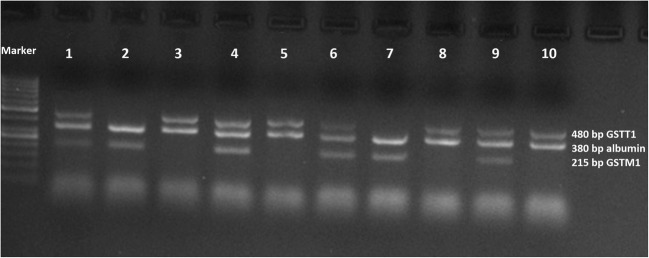
Electrophoretic separation of DNA fragments obtained by multiplex PCR, showing *GSTM1* and *GSTT1* deletion in the studied samples (lanes 1, 4, 6, and 9, patients with *GSTM1+/GSTT1+* variant; lanes 2 and 7, patients with *GSTT1* deletion; lanes 3, 5, 8, and 10, patients with *GSTM1* deletion), pBR322 DNA/Alu I—DNA size marker

**Fig. 2:**
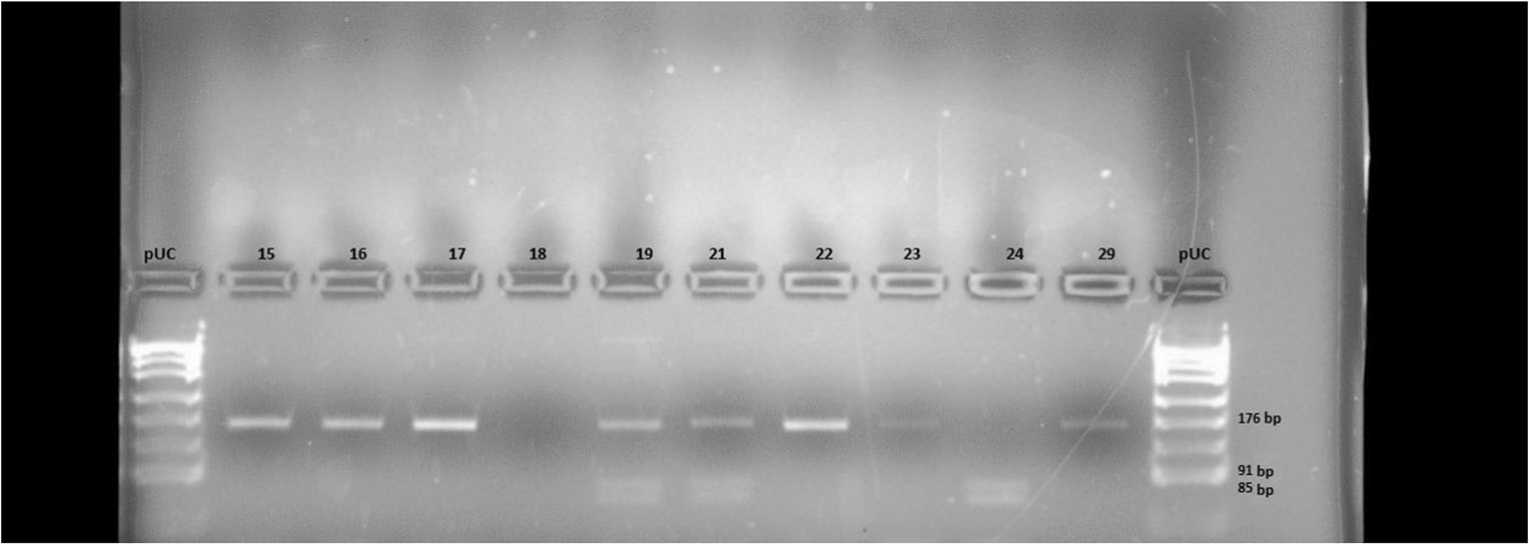
Electrophoretic separation of DNA fragments obtained by PCR-RFLP, showing the presence of polymorphism *Ile/Ile, Ile/Val*, and *Val/Val GSTP1* in the studied samples (lanes 15, 16, 17, 22, 23, and 29, patients with the *GSTP1 Ile/Ile* genotype; lanes 19 and 21, patients with the *Ile/Val GSTP1* genotype; lane 24, a patient with the genotype *GSTP1 Val/Val*), pUC/MspI—DNA size marker

The results of molecular analysis of glutathione S-transferase polymorphisms were obtained in the form of digital images of electrophoretic gels for polymorphisms *GSTM1* and *GSTT1* (Fig. 1) and separately for the *GSTP1* polymorphism (Fig. 2).

**Fig. 3:**
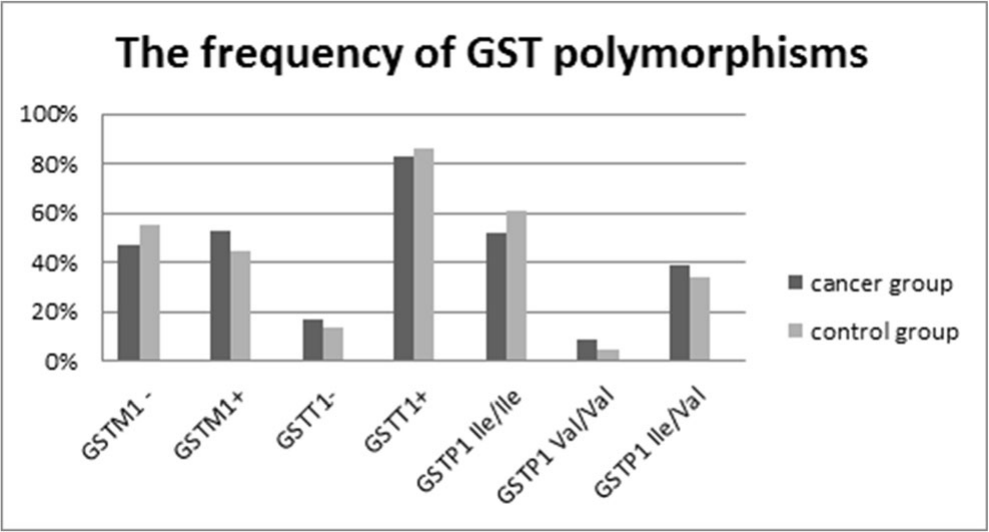
Frequency of *GSTM1*, *GSTT1*, and *GSTP1* polymorphisms in the cancer group (*n* = 66) and in the control (*n* = 64) “*GSTM1+*,*”* no deletion in the gene; “*GSTM1−*,*”* deletion of GSTM1; “*GSTT1+*,*”* no deletion in the gene; “*GSTT1−*,*”* deletion of GSTT1

The results obtained by electrophoresis corresponded to a genotype present in the cancer group and in the control. There were no statistically significant differences between the groups for any of the three genotypes tested (Table 1; Fig. 3).

**Table 1:** Frequency of *GSTM1*, *GSTT1*, and *GSTP1* polymorphisms in the cancer group (*n* = 66) and in the control (*n* = 64)

Genotype		Cancer group		Control group		*p*
		*N*	%	*N*	%	
GSTM1 genotype	–	31	46.97%	35	54.69%	0.378
	+	35	53.03%	29	45.31%	
GSTT1 genotype	–	11	16.67%	9	14.06%	0.68
	+	55	83.33%	55	85.94%	
GSTP1 genotype	Ile/Ile	34	51.52%	39	60.94%	0.439
	Val/Val	6	9.09%	3	4.69%	
	Ile/Val	26	39.39%	22	34.38%

## Discussion

Prostate cancer is currently one of the most commonly diagnosed cancers in men from developed countries. It is likely that its incidence will increase with increasing age of the population. Mechanisms of prostate carcinogenesis, involving both genetic and environmental factors, remain unclear. Changes in genes of DNA repair pathways that play a critical role in the prevention of carcinogenesis may lead to a higher susceptibility to cancer, including prostate cancer (Gong et al. 2012; Cai et al. 2014; Shin et al. 2016).

Glutathione S-transferases are superfamily of enzymes of the second phase of xenobiotic metabolism that play an important role in the genetics of prostate cancer. These enzymes catalyze the conjugation of chemically reactive compounds with reduced glutathione. They also protect DNA and other macromolecules from damage caused by oxidative stress. It is believed that the GSTM1 enzyme is involved in the metabolism of styrene oxide, the GSTT1 enzyme plays a role in the detoxification of dichloromethane and ethylene oxide, while the GSTP1 enzyme is responsible for the detoxification of carcinogenic heterocyclic amines. All these substances are probable carcinogens; therefore, the impaired function of the above-mentioned enzymes does not allow the efficient removal of toxic compounds, which indirectly may increase the susceptibility to various cancers, including prostate cancer. However, the mechanisms by which polymorphisms of the glutathione S-transferase can increase the risk of prostate cancer are still unknown (Ntais et al. 2005; Cai et al. 2014; Chirilă et al. 2015; Qadri et al. 2011; Saheb et al. 2016).

The goal of this study was to determine whether the *GSTM1, GSTT1*, and *GSTP1* polymorphisms can modify the risk of developing prostate cancer. We presented the results of the first-ever analysis of the frequency of glutathione S-transferase polymorphism in the group of Polish patients with prostate cancer and in a properly selected control group.

Statistical analysis of the results did not show statistically significant differences in the frequency of the polymorphisms between the analyzed groups. The deletion of *GSTM1* was found in ca. 47% of patients with prostate cancer and in ca. 55% of controls. The *GSTT1* deletion was found in ca. 17% of patients and ca. 14% of controls. In turn, the distribution of *GSTP1 Ile/Ile, Ile/Val*, and *Val/Val* polymorphisms was ca. 51.5%, 39%, and 9% in the study group and 61%, 34%, and 5% in the control, respectively.

Mo et al. (2009) in their meta-analysis reported the frequency of *GSTM1* deletions in the range from 13.1 to 54.5% and the *GSTT1* null polymorphism from 11.1 to 28.6%. These authors provided previously published data on the frequency of the *Val/Val* allele, from 9 to 12.6% in Caucasians, and also reported a higher percentage (38.9–62.1%) obtained in their own study; the latter results may be distorted by including studies on combined variations *Ile/Val* and *Val/Val*. According to these data, the frequency of the *GSTM1* and *GSTT1* polymorphisms reported in our study for both the study group and the control are within the range found in the general population. It is interesting that a higher percentage of GSTM1 deletions was observed in the control group (55%), while in the study group, it was slightly lower (47%). However, the difference was not statistically significant and might be due to the small number of people participating in the study, because each group contained less than 70 patients. In addition, no data on the frequency of GSTM1 deletion in the Polish population was available until now, which made it impossible to compare our results with the corresponding general population. The *GSTP1 Val/Val* frequency in the study group falls within the range previously observed in Caucasians, while in the control, the percentage is lower than expected, which was caused by a lower number of participants than in other studies. It should also be noted that similar low frequencies of the *GSTP1 Val/Val* allele in Caucasians were reported by other authors, e.g., Mittal and co-workers 2006, 4.8%; or Srivastava and co-workers 2005, 3.5%.

In their meta-analysis, Mo and co-workers 2009 found that the *GSTM1* deletion increased the risk of prostate cancer in the general population, including Asians, Afro-Americans, and populations of African and Caucasian descent. In the case of the *GSTT1* and *GSTP1* polymorphisms, there were no statistically significant relationships with the disease (Mo et al. 2009); it was established that the polymorphisms are not related to the risk of prostate cancer.

Another meta-analysis by Gong and co-workers 2012 showed a significant association of *GSTM1* polymorphism with the risk of prostate cancer; however, it did not find such correlations for the *GSTT1* and *GSTP1* deletions, thus confirming the results of Mo and co-workers 2009. Additionally, Gong and co-workers 2012 examined the combination of both genotypes and showed a significant increase in the risk of disease in men with double-null genotype *GSTM1−/GSTT1−* and those with *GSTT1-/GSTP1 Ile/Val*. In this study, no comparison of combined phenotypes (*GSTM1/T1/P1*) was made due to the low number of men in the resulting subgroups making it impossible to obtain statistically significant results.

The latest meta-analysis by Cai and co-workers 2014 also showed significant relationships between prostate cancer and *GSTM1* polymorphism in Caucasians and the lack of such correlations for *GSTT1* allele. This meta-analysis did not include the study of genetic variants of glutathione S-transferase GSTP1. The latest reports on this polymorphism, cited in the meta-analysis of Wei and co-workers 2013, indicate a lack of dependence between *GSTP1 Ile/Ile*, *Ile/Val*, and *Val/Val* variants and an increased risk of prostate cancer. In general, these studies show the same results, but they also mention numerous limitations, such as the size of the study groups, their heterogeneity, or the lack of analysis for some populations. For example, to our knowledge, no studies have been conducted so far in Polish population on general frequency of polymorphisms of glutathione S-transferase and the relationships of these genetic changes with prostate cancer (cf. Mo et al. 2009; Gong et al. 2012; Cai et al. 2014).

The results presented in this paper, showing the frequency of genetic variants *GSTM1*, *GSTT1*, and *GSTP1* confirm the lack of connection of glutathione S-transferase *T1* and *P1* polymorphisms with the risk of prostate cancer; however, they do not support claims that *GSTM1* genetic variants may play a role in increasing the risk of this cancer. It should be noted that these are the first studies of this type, including Polish patients with prostate cancer, comparing the results with a properly selected control group.

This study showed that there was no relationship between glutathione S-transferase polymorphisms and prostate cancer in the analyzed group of patients, which makes our results different from those obtained in previous studies on the role of these genetic variants in cancer etiology.
